# A cost-effective, high-throughput, highly accurate genotyping method for outbred populations

**DOI:** 10.1093/g3journal/jkae291

**Published:** 2024-12-13

**Authors:** Denghui Chen, Apurva S Chitre, Khai-Minh H Nguyen, Katerina A Cohen, Beverly F Peng, Kendra S Ziegler, Faith Okamoto, Bonnie Lin, Benjamin B Johnson, Thiago M Sanches, Riyan Cheng, Oksana Polesskaya, Abraham A Palmer

**Affiliations:** Bioinformatics and System Biology Program, University of California San Diego, La Jolla, CA 92093, USA; Bioinformatics and System Biology Program, University of California San Diego, La Jolla, CA 92093, USA; Department of Psychiatry, University of California San Diego, La Jolla, CA 92093, USA; Department of Psychiatry, University of California San Diego, La Jolla, CA 92093, USA; Department of Psychiatry, University of California San Diego, La Jolla, CA 92093, USA; Department of Psychiatry, University of California San Diego, La Jolla, CA 92093, USA; Department of Psychiatry, University of California San Diego, La Jolla, CA 92093, USA; Department of Psychiatry, University of California San Diego, La Jolla, CA 92093, USA; Department of Psychiatry, University of California San Diego, La Jolla, CA 92093, USA; Department of Psychiatry, University of California San Diego, La Jolla, CA 92093, USA; Department of Psychiatry, University of California San Diego, La Jolla, CA 92093, USA; Department of Psychiatry, University of California San Diego, La Jolla, CA 92093, USA; Department of Psychiatry, University of California San Diego, La Jolla, CA 92093, USA; Institute for Genomic Medicine, University of California San Diego, La Jolla, CA 92093, USA

**Keywords:** low-coverage, whole-genome sequencing, genotyping, heterogeneous stock rat

## Abstract

Affordable sequencing and genotyping methods are essential for large-scale genome-wide association studies. While genotyping microarrays and reference panels for imputation are available for human subjects, nonhuman model systems often lack such options. Our lab previously demonstrated an efficient and cost-effective method to genotype heterogeneous stock rats using double-digest genotyping by sequencing. However, low-coverage whole-genome sequencing offers an alternative method that has several advantages. Here, we describe a cost-effective, high-throughput, high-accuracy genotyping method for N/NIH heterogeneous stock rats that can use a combination of sequencing data previously generated by double-digest genotyping by sequencing and more recently generated by low-coverage whole-genome sequencing data. Using double-digest genotyping-by-sequencing data from 5,745 heterogeneous stock rats (mean 0.21× coverage) and low-coverage whole-genome sequencing data from 8,760 heterogeneous stock rats (mean 0.27× coverage), we can impute 7.32 million biallelic single-nucleotide polymorphisms with a concordance rate > 99.76% compared to high-coverage (mean 33.26× coverage) whole-genome sequencing data for a subset of the same individuals. Our results demonstrate the feasibility of using sequencing data from double-digest genotyping by sequencing or low-coverage whole-genome sequencing for accurate genotyping and demonstrate techniques that may also be useful for other genetic studies in nonhuman subjects.

## Introduction

In both humans and model organisms, genome-wide association studies (GWAS) are valuable for identifying genetic variants associated with diseases and other complex traits. GWAS results facilitate the discovery of novel biological pathways and potential therapeutic targets (Palmer *et al*. 2021; Uffelmann *et al*. 2021; Alliance of Genome Resources Consortium 2022; Abdellaoui *et al*. 2023). The success of large-scale population and quantitative genetics studies depends on the availability of dense and high-quality genotype data (Welter *et al*. 2014). Single-nucleotide polymorphism (SNP) arrays, paired with reference panels (e.g. HapMap or the 1000 Genomes Project), are commonly used to infer genotypes and perform genetic studies in humans (Frazer *et al*. 2007; Marchini and Howie 2010; McVean *et al*. 2012; Uffelmann *et al*. 2021; Aganezov *et al*. 2022). However, SNP arrays often perform poorly when applied to populations other than the one used for array design, leading to a need for costly development of population-specific SNP arrays (Didion *et al*. 2012). This issue is even more critical in model organisms, where population structure is often very pronounced (Gileta *et al*. 2022). An alternative to genotyping microarrays is to use next-generation sequencing. Because sequencing at sufficient depth to make calls directly remains expensive, low-coverage sequencing paired with imputation from reference panels provides a more economical solution (Davies *et al*. 2016; Petter *et al*. 2020; Li *et al*. 2021, 2024; Wasik *et al*. 2021).

Our lab has performed GWAS using various mouse and rat populations (Chitre *et al*. 2020, 2023; Zhou *et al*. 2020; Gileta *et al*. 2022; Gunturkun *et al*. 2022; Parker *et al*. 2022; Fowler *et al*. 2023). In particular, we have now phenotyped and genotyped almost 20,000 N/NIH heterogeneous stock (HS) rats. HS rats were created in 1984 by intercrossing 8 inbred rat strains (ACI/N, BN/SsN, BUF/N, F344/N, M520/N, MR/N, WKY/N, and WN/N). To genotype outbred mice and rats, we have used genotyping by sequencing (GBS) (Elshire *et al*. 2011; Parker *et al*. 2016; Gonzales *et al*. 2018) and subsequently double-digest GBS (ddGBS) protocols, followed by imputation (Gileta *et al*. 2020). More recently, we have reported on our use of commercial whole-genome sequencing (WGS) library preparation kits to generate low-coverage WGS (lcWGS) data, followed by imputation using outbred mice (Davies *et al*. 2016; Nicod *et al*. 2016; Zou *et al*. 2022). However, we have not previously reported on our methods for genotyping rats using lcWGS followed by imputation, nor have we reported a method for jointly calling genotypes using a combination of ddGBS and lcWGS data.

In this paper, we present a cost-effective, high-throughput, and highly accurate genotyping method for HS rats that utilizes both previously generated ddGBS data and more recently generated lcWGS data. This method allowed us to impute 7.32 million biallelic SNPs with a concordance rate of >99.76% compared to genotypes obtained from 33.26× coverage WGS without imputation for a subset of the same individuals.

## Materials and methods

### Animals

As reviewed elsewhere, the N/NIH HS rat population was created by interbreeding 8 inbred rat strains (ACI/N, BN/SsN, BUF/N, F344/N, M520/N, MR/N, WKY/N, and WN/N) in the mid-1980s (Solberg Woods and Palmer 2019). Since then, HS rats have been maintained as an outbred population for more than 100 generations. Because they have been maintained as an outbred population for such a long time, HS rats possess short haplotypes that are derived from the 8 inbred founders, making them ideal for high-resolution genetic mapping (Johannesson *et al*. 2009; Baud *et al*. 2013; Woods and Mott 2017; Solberg Woods and Palmer 2019). In this study, we used sequence data from a total of 15,552 HS rats (7,797 males and 7,755 females) from generation 81 to 97 that were bred at the Medical College of Wisconsin (RRID: RGD_2314009), Wake Forest University (RRID: RGD_13673907), the University of Tennessee Health Sciences Center, or Oregon Health and Sciences University. The colony at the Medical College of Wisconsin moved to Wake Forest University in 2016, which resulted in 2 sites that existed sequentially. The University of Tennessee Health Sciences Center and Oregon Health and Sciences University bred rats from Wake Forest University for a single generation to produce offspring locally; therefore, RRIDs have not been issued for these 2 sites. Detailed composition of rats by sex and site is outlined in Supplementary Table 1. All procedures that occurred prior to tissue collection were approved by the relevant Institutional Animal Care and Use Committees. As described in the following sections, of the 15,552 HS rats, 477 were sequenced with both ddGBS and lcWGS. Eighty-eight of those 477 were also whole-genome sequenced at an average depth of 33.26×; we refer to those 88 rats as the “truth set.”

### ddGBS sequencing

Of the 15,552 HS rats used in this study, 6,379 individuals (3,219 males and 3,160 females) were sequenced using a ddGBS library preparation protocol described by (Gileta *et al*. 2020). Briefly, DNA was extracted from spleen tissues using Agencourt DNAdvance Kit (Beckman Coulter Life Sciences, Indianapolis, IN, USA) and digested using the restriction enzymes Pstl and NlaIII. After adapter ligation, DNA purification, and library pooling, sample DNA was sequenced as 48 samples per library on Illumina HiSeq 4000 with 100-bp single-end reads at the University of California San Diego Institute for Genomic Medicine Genomics Center (UCSD IGM).

### lcWGS sequencing

In addition, 9,173 (4,578 males and 4,595 females) of 15,552 HS rats underwent lcWGS sequencing. DNA was extracted from spleen tissues using the Agencourt DNAdvance Kit, and the Twist 96-Plex Library Prep Kit (Twist Bioscience, South San Francisco, CA, USA) was used for library preparation following the manufacturer's protocol. In each library, 96 samples were barcoded separately. Then, the samples’ DNA was sequenced on Illumina NovaSeq 4000 or 6000 with 150-bp paired-end reads at UCSD IGM. DNA extraction, normalization, randomization, and library preparation were all performed on the EPmotion 5075 (Eppendorf, Hamburg, Germany) liquid-handling robot. Detailed lcWGS protocols for many of these steps can be found in the Center for GWAS in Outbred Rats Database protocol repository on protocols.io (https://www.protocols.io/workspaces/cgord, spleen cutting: http://dx.doi.org/10.17504/protocols.io.36wgq7nryvk5/v1, DNA extraction: http://dx.doi.org/10.17504/protocols.io.8epv59reng1b/v1, normalization and randomization: http://dx.doi.org/10.17504/protocols.io.261genw5dg47/v1, library preparation: http://dx.doi.org/10.17504/protocols.io.j8nlkkm85l5r/v1, pooling and sequencing: http://dx.doi.org/10.17504/protocols.io.yxmvmnw29g3p/v1).

### Reference panel preparation

To obtain the best possible imputation reference panel for outbred HS rats, we used consensus biallelic homozygous SNP calls from 3 different inbred HS rat founder data sets. The first data set was produced from publicly available 30.34× coverage WGS sequences (NCBI SRA: PRJNA487943) using the Genome Analysis Toolkit (GATK) joint calling workflow (Supplementary Method 2 and Supplementary Fig. 1) (Ramdas *et al*. 2019; Van der Auwera and O’Connor 2020). In that data set, BN/SsN and MR/N are female, and other rats are male. The second data set was produced using the same GATK joint calling workflow using an independent data set with an average of 41.81× coverage WGS sequences (NCBI SRA: PRJNA1048943) generated with high-coverage WGS sequencing procedures (Supplementary Methods 1 and 2 and Supplementary Fig. 1). Details of this data set have not been previously published. In this data set, all 8 HS founders were male. The third data set was produced using the same 41.81× coverage WGS sequences, but using the DeepVariant multisample calling workflow (Supplementary Method 3 and Supplementary Fig. 2). Filters applied after variant calling processes were described in the corresponding supplementary method sections. For autosomal chromosomes, chromosome X, and mitochondria, 7,406,667, 184,934, and 117 SNPs respectively that had consensus homozygous genotypes across all 3 call sets were retained; however, because BN/SsN and MR/N in the first data set are female, we dropped them from the consensus check process for chromosome Y, resulting in 5,220 consensus homozygous SNPs for chromosome Y. In total, 7,596,938 SNPs were retained for the reference panel.

### Biallelic SNP positions preparation

We employed STITCH for the imputation process. STITCH was designed for imputing biallelic SNPs in lcWGS reads by constructing haplotypes (Davies *et al*. 2016). STITCH accepts a position file for the biallelic SNPs to be imputed. In order to capture the common variants derived from the HS founders, as well as new SNPs observed in recent generations of the outbred HS population, we compiled the SNP position file using biallelic SNPs discovered in the founder data sets mentioned above and in 88 HS rats (44 males and 44 females). Variants in the subset of 88 HS rats were called on 33.26× coverage WGS sequences (NCBI SRA: PRJNA1076141) using the GATK joint calling workflow (Supplementary Methods 1 and 2 and Supplementary Fig. 1). The resulting SNPs position file contained 10,684,883 SNPs with 10,227,209 on autosomal chromosomes, 331,389 on chromosome X, 126,141 on chromosome Y, and 144 on mitochondria.

### Truth set preparation

To assess the quality of imputed genotypes, we sequenced the aforementioned 88 HS outbred rats using 3 methods: ddGBS, lcWGS, and high-coverage WGS (33.26×). The biallelic SNPs imputed from the ddGBS and lcWGS genotyping pipeline were compared with the variants discovered on high-coverage WGS GATK joint calling pipeline (Supplementary Methods 1 and 2 and Supplementary Fig. 1). Variants filtering process was described in the supplementary method as well. We treated the genotypes called by high-coverage WGS as our truth set and used them to check the concordance of the genotypes imputed with the other 2 methods.

### Genotyping

**Fig. 1.**
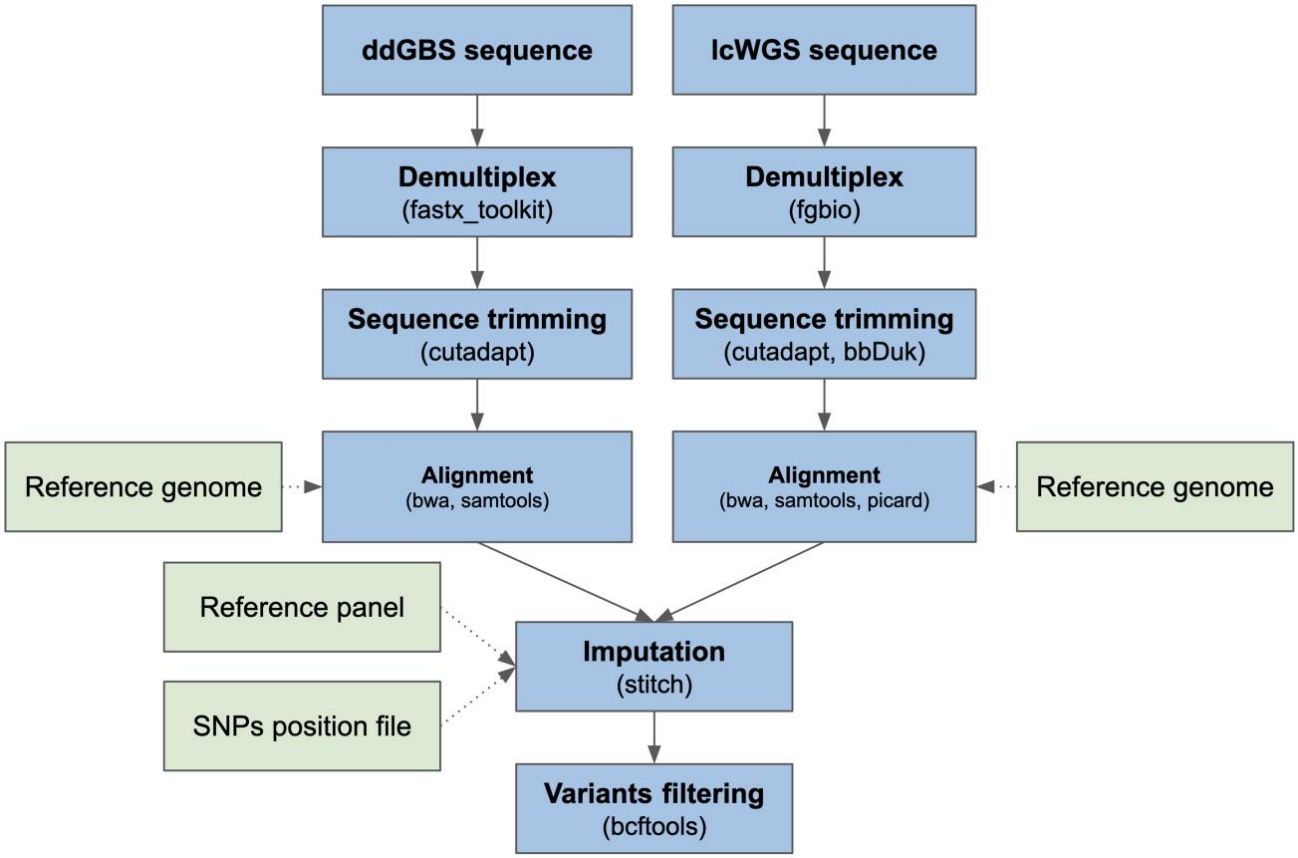
Genotyping pipeline flow chart.

Our full bioinformatic pipeline is outlined in Fig. 1. The pipeline inputs each sample's raw ddGBS or lcWGS sequences, maps them to *Rattus norvegicus* reference genome mRatBN7.2 (NCBI Genome Assembly Accession: GCF_015227675.2) in parallel, and then jointly imputes biallelic SNPs. The complete source code for the pipeline can be found in the Palmer Lab GitHub repository (https://github.com/Palmer-Lab-UCSD/HS-Rats-Genotyping-Pipeline, DOI:https://doi.org/10.5281/zenodo.10002191).

ddGBS sequences were demultiplexed using fastx_toolkit v0.0.14 (Hannon Lab 2010). Barcode, adapter, and quality trimming were subsequently performed using Cutadapt v4.1 (Martin 2011) with 25 bp as the minimum length per read and 20 as the minimum base quality. BWA-mem v0.7.17 (Li 2013) was used to align ddGBS sequences with a constraint of an alignment score greater than 20, and the aligned BAM files were sorted and indexed by coordinates using SAMtools v1.14 (Danecek *et al*. 2021) for fast random access.

lcWGS sequences were demultiplexed using fgbio v1.3.0 (Tim and Nils 2023). BBDuk v38.94 (Bushnell 2024) (ktrim = r, k = 23, mink = 11, hdist = 1, trimpolyg = 50, tpe, tbo) was used to trim adapters, and Cutadapt v4.1 (Martin 2011) was used to trim sequences with Phred base quality < 5 and length shorter than 70 bp. Alignment of the lcWGS sequences was carried out using BWA-mem v0.7.17 (Li 2013). Duplicated reads were marked using Picard v2.25.7 (Broad Institute 2019) and indexed by coordinates using SAMtools v1.14 (Danecek *et al*. 2021) for fast random access.

Aligned sequences were used to jointly impute biallelic SNPs at given positions with STITCH v1.6.6 (Davies *et al*. 2016) (niterations = 2, k = 8, nGen = 100). At the first iteration of STITCH's EM algorithm, the reference haplotypes are used to initialize the ancestral haplotype. After the first iteration, STITCH uses information from the samples’ reads to update the ancestral haplotypes. In our genotyping pipeline, we set *niterations* parameter to 2 to enable STITCH to capture variants not present in the provided reference panel. Since the HS rat population was derived from 8 inbred founders, we set the STITCH *k* parameter to 8 to specify the number of founder haplotypes to use. STITCH also requires an *nGen* parameter for the number of population generations. For the results presented in this paper, we used 100. We experimented with other values and found that this parameter had virtually no impact on our results. During the imputation step, a reference panel based on the genotypes of the 8 inbred founder strains and the SNP position file mentioned above were provided to STITCH to construct haplotypes for imputation. To increase computational efficiency, imputation was performed parallelly on chromosome chunks with a 1-Mb buffer on each end. Each chunk had a length of at least 7 Mb and contained at least 1,000 SNPs. Then, we used BCFtools v1.14 (Danecek *et al*. 2021) to concatenate the chunks back to individual chromosomes.

### SNP quality control

Following the imputation process, we implemented a quality control procedure to filter out SNPs with low genotype quality. A total of 10,684,883 biallelic SNPs were imputed using our genotyping pipeline. Among them, we removed 2,737,742 SNPs with an imputation info score < 0.9 using BCFtools v1.14 (Danecek *et al*. 2021). Furthermore, we filtered out 623,881 SNPs that have low concordance with the ground truth data set described above. As a result, we retained 7,323,260 SNPs. The genotypes after quality control can be found in UC San Diego Library Digital Collections (https://doi.org/10.6075/J0445MPC).

### Sample quality control

A sample quality control step was also performed to ensure sample quality. In total, 15,552 samples, representing 14,629 unique outbred HS rats, were used in this study. We excluded 66 samples whose ratio of mapped reads on chromosomes X and Y were incompatible with their reported sex (Supplementary Fig. 3). We also filtered out samples with high genotype missing rate and samples with possible contamination based on their genotype heterozygosity rate. Specifically, we excluded 153 samples that either had a genotype missing rate exceeding 0.1 or a genotype heterozygosity rate falling outside the range of ±4 SD (Supplementary Fig. 4). Because of the differences between ddGBS and lcWGS data, we conducted these 2 sample quality control criteria for different sequencing methods separately. Additionally, in the cases where we had multiple sequencing runs for the same samples, we kept only the one with the highest number of sequence reads. This quality control process resulted in the retention of 14,505 distinct HS rats (7,283 males and 7,222 females) with 5,745 individuals from ddGBS (2,903 males and 2,842 females) and 8,760 individuals from lcWGS (4,380 males and 4,380 females).

## Results

### Sequence statistics

Our genotyping pipeline was applied to 15,552 samples, representing 14,629 unique outbred HS rats. A total of 14,505 distinct samples were retained after the quality control steps described in *Materials and Methods* section, 5,745 of which were sequenced using ddGBS and 8,760 using lcWGS.

**Fig. 2.**
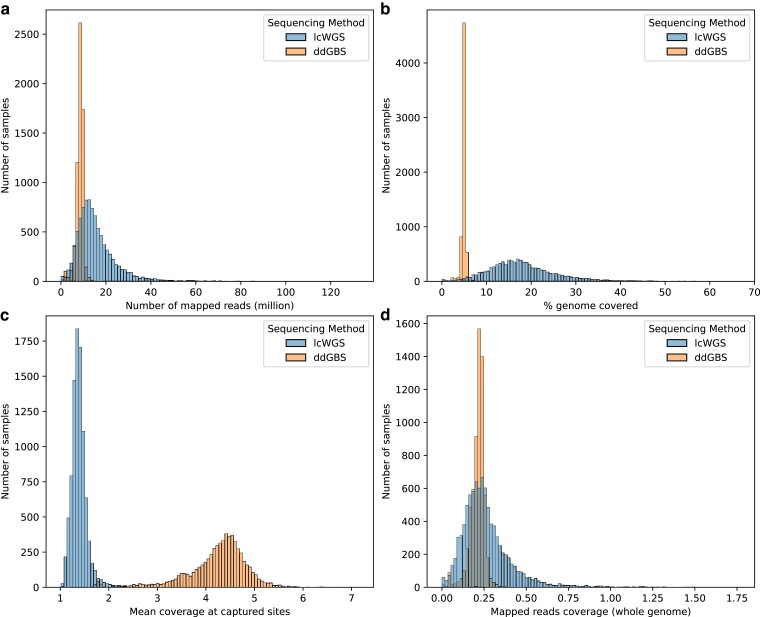
Aligned sequence statistics. a) Number of reads mapped to reference genome (million). ddGBS mean: 8.44, SD: 1.65; lcWGS mean: 16.03, SD: 10.32. b) Percentage of genome covered by mapped reads in width (%). ddGBS mean: 4.97, SD: 0.54; lcWGS mean: 18.28, SD: 8.31. c) Mean coverage at captured sites. ddGBS mean: 4.22×, SD: 0.67×; lcWGS mean: 1.39×, SD: 0.16×. d) Mapped reads coverage genome wide. ddGBS mean: 0.21×, SD: 0.04×; lcWGS mean: 0.27×, SD: 0.16×.

After demultiplexing and aligning to reference genome mRatBN7.2 (NCBI Genome Assembly Accession: GCF_015227675.2), a mean of 8.44 million 100-bp reads per sample was mapped to the reference genome in the case of ddGBS (Fig. 2a). Because of the double restriction enzyme digestion employed in ddGBS, only the chromosomal regions near the enzyme cut sites were sequenced. This led to ddGBS sequences covering 4.97% of the genome per sample, with a mean coverage of 4.22× at each captured site (Fig. 2b and c). Consequently, this approach resulted in an average mapped coverage of 0.21× per sample across the entire genome although that coverage was highly nonuniform, by design (Fig. 2d).

For lcWGS, a mean of 16.03 million 150-bp reads were mapped for each sample (Fig. 2a). Due to the random priming process of lcWGS, a more diverse set of DNA fragments was sequenced. This enabled lcWGS sequences to cover a wider range of the genome at 18.28% per sample on average, but with a lower mean coverage of 1.39× at each capture site (Fig. 2b and c). This resulted in a mean mapped coverage of 0.27× per sample genome wide (Fig. 2d).

### Genotype statistics

**Fig. 3.**
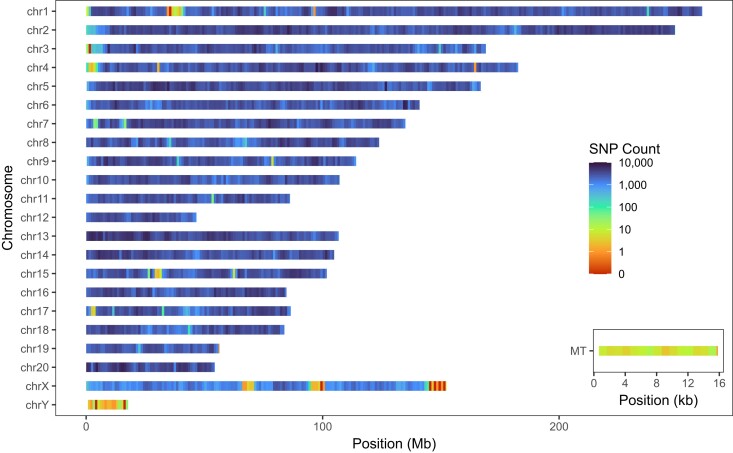
Imputed biallelic SNPs density distribution heatmap on each chromosome with 1-Mb windows and mitochondrial chromosome with 1-kb windows.

In our genotyping pipeline, we imputed a total of 10,684,883 biallelic SNPs. Following the quality control procedures outlined in *Materials and Methods* section, 7,323,260 SNPs were retained. Out of these retained SNPs, 7,148,654 were located on autosomal chromosomes, 174,374 were on chromosome X, 118 were on chromosome Y, and 114 were on mitochondria (Fig. 3).

Among the 7,148,654 SNPs on autosomes, 1,602,374 were found to be monomorphic with a minor allele frequency (MAF) of 0. We assume that these SNPs, which were polymorphic in the HS founders, became monomorphic in the outbred HS population due to genetic drift, the proportion of SNPs showing this pattern is consistent with simulations we have performed in the past (Munro *et al*. 2022). Additionally, new mutations may have arisen since the creation of the HS population, most of which are expected to have low MAF. The primary objective of our SNP genotyping is to identify variants useful for GWAS; however, low MAF SNPs are not well powered to detect associations. Therefore, we applied a MAF threshold of 0.005. A total of 183,621 SNPs fell below the MAF ≤ 0.005 threshold whereas 5,362,659 were above the threshold (Supplementary Fig. 5a). Further examination revealed that 143,402 of the SNPs with a MAF ≤ 0.005 had an allele count lower than 10, and 136,542 had an allele count lower than 5 (Supplementary Fig. 8a), suggesting that many of the low MAF SNPs were genotyping errors. In this study, all the HS rats used were from the same population; however, familial relationships within the colony could lead to deviations from Hardy–Weinberg equilibrium (HWE) that would not justify excluding the SNPs. Therefore, we applied a lenient HWE threshold of −log10(*P*-value) < 10. Out of the 7,148,654 SNPs, 39,606 violated HWE with a −log10(*P*-value) ≥ 10 (Supplementary Fig. 5b), and 36,664 had a genotype missing rate higher than 0.1 (Supplementary Fig. 5c). Consequently, a total of 5,292,916 autosomal SNPs had a MAF > 0.005, HWE −log10(*P*-value) < 10 and missing rate ≤ 0.1.

### Sex chromosomes

Due to the different inheritance patterns on sex chromosomes in males and females, we investigated the SNPs on chromosomes X and Y separately in each sex. Among the 7,222 female samples included in this study, we observed that out of the 174,374 SNPs on chromosome X, 47,882 were monomorphic, 1,375 had a MAF ≤ 0.005, and 125,117 had a MAF > 0.005 (Supplementary Fig. 6a). A total of 627 SNPs violated HWE (Supplementary Fig. 6b), and 582 SNPs had a missing rate higher than 0.1 (Supplementary Fig. 6c). This led to a total of 123,997 chromosome X SNPs for females with a MAF > 0.005, HWE −log10(*P*-value) < 10 and missing rate ≤ 0.1. Chromosome Y SNPs were discarded for female samples. In the 7,283 male samples used in this study, among the 174,374 SNPs on chromosome X, 46,319 were monomorphic, 3,227 had a MAF ≤ 0.005, and 124,828 had a MAF > 0.005 (Supplementary Fig. 6d). Because males have only one copy of the X chromosome, we did not test them for HWE, but we found 2,223 chromosome X SNPs had a missing rate higher than 0.1 (Supplementary Fig. 6e). This resulted in a total of 122,693 chromosome X SNPs for males with a MAF > 0.005 and missing rate ≤ 0.1. The 118 SNPs on chromosome Y for male samples had a missing rate ≤ 0.1, but they were all monomorphic SNPs with a MAF of 0.

Out of the 114 SNPs on the mitochondrial chromosome, 30 were found to be monomorphic with a MAF of 0, and the remaining 74 were SNPs with MAF > 0.005 (Supplementary Fig. 7a). HWE was also not tested for mitochondrial SNPs, but all of them had a genotype missing rate lower than 0.1 (Supplementary Fig. 7b). Consequently, a total of 74 mitochondria SNPs had a MAF > 0.005 and missing rate ≤ 0.1.

We have recently published a separate paper that uses the same genotypes described here to examine Y and mitochondrial chromosome haplogroups (Okamoto et al. 2024).

### Genotype accuracy

**Fig. 4.**
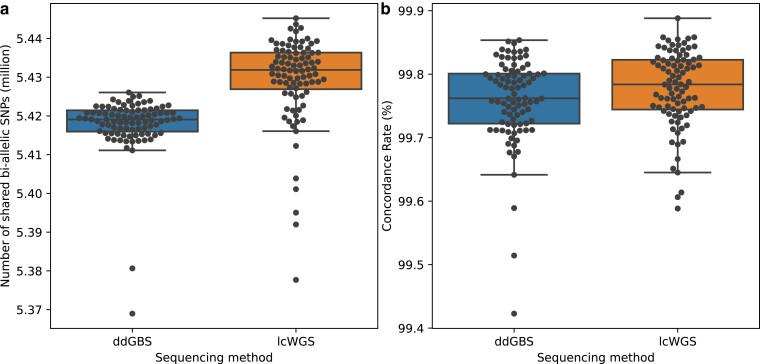
Imputed genotypes demonstrate high concordance with 33.26× high-coverage WGS for millions of biallelic SNPs. a) Number of biallelic SNPs compared (million). ddGBS mean: 5.42, SD: 0.01; lcWGS mean: 5.43, SD: 0.01. b) Concordance rate with 33.26× high-coverage WGS (%). ddGBS mean: 99.76, SD: 0.07; lcWGS mean: 99.78, SD: 0.06.

As described in *Materials and Methods* section, in the 15,552 outbred HS rats, we genotyped, there were 88 outbred HS rats that had been sequenced with ddGBS, lcWGS, and 33.26× high-coverage WGS. We tested our genotyping pipeline's accuracy by comparing genotypes imputed from ddGBS and lcWGS with SNPs called using high-coverage WGS without any imputation, which we refer to as the “truth set.” Specifically, for each sample, we looked at the concordance rate of overlap and nonmissing SNPs between the imputed genotypes and the truth set. Concordance rate calculations were based on SNPs that passed quality control filters: MAF > 0.005, HWE −log10(*P*-value) < 10, and missing rate ≤ 0.1. On average, 5,417,913 polymorphic SNPs were shared between imputation from ddGBS sequences and variant calling from 33.26× high-coverage WGS, with a mean concordance rate of 99.76% (Fig. 4). Similarly, we observed that 5,429,453 SNPs were shared between lcWGS and 33.26× high-coverage WGS, with a mean concordance rate of 99.78% (Fig. 4). Additionally, we examined the concordance across different MAFs. SNPs at different MAFs were relatively uniformly distributed. The genotype concordance rate started at around 99.98% and decreased slightly as MAF increased such that accuracy dropped to about 99.6% as MAF approached 0.5. Overall these results indicate a high concordance across all allele frequencies (Supplementary Fig. 9). ddGBS sequences a smaller portion of the genome at higher depth, while lcWGS covers a larger portion at lower depth. These differences lead to more regions without reads in ddGBS compared to lcWGS, resulting in a slightly higher number of discordant calls in those regions (Supplementary Fig. 10). For the imputed genotypes on chromosome X, the concordance rates from 2 sequencing approaches are comparable (Supplementary Fig. 11).

### Batch effects on ddGBS and lcWGS genotypes

**Fig. 5.**
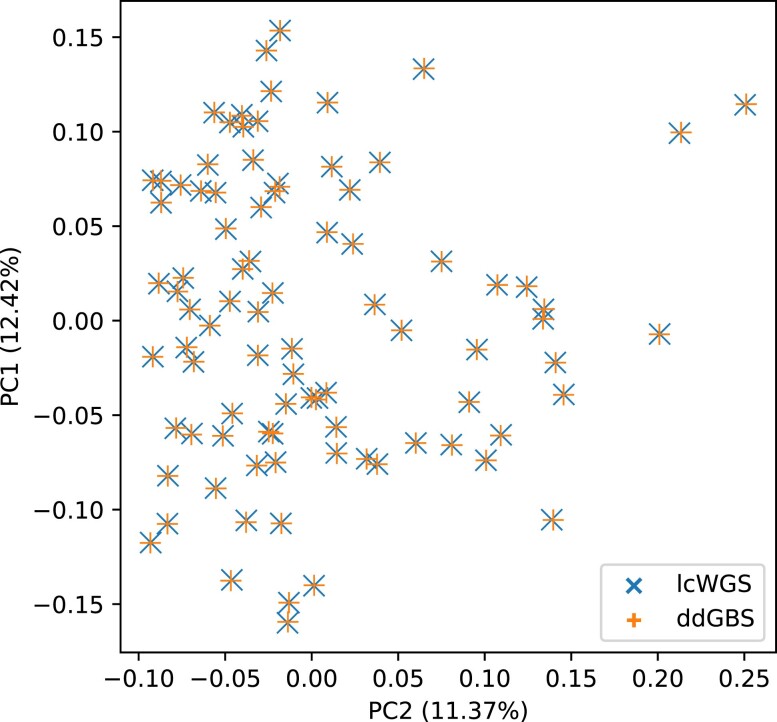
Overlapping first and second PCs on genotypes shows no batch effects between different sequencing methods.

To investigate potential batch effects of different sequencing methods, we performed a principal component (PC) analysis on the autosomal genotypes of the 88 HS outbred rats sequenced with both ddGBS and lcWGS (Fig. 5). Overlapping first and second PC values without apparent clustering between the 2 methods indicate that both methods capture equivalent information from the genome, meaning there are no obvious method-specific batch effects introduced by the pipeline. Additionally, we did not observe any batch effects in any other PCs that explained more than 10% of the variance (Supplementary Fig. 12). The MAF and HWE distributions were also comparable across different sequencing batches with minor indication of genetic drift on MAF (Supplementary Fig. 13).

## Discussion

While large-scale genetic studies in humans often use genotyping microarrays and imputation, similar resources are not available for most other species. Although there are examples where human genetic studies use lcWGS and imputation for genotyping, they typically require higher coverage because of more diverse and smaller haplotype blocks (Cai *et al*. 2015; Petter *et al*. 2020; Li *et al*. 2021, 2024; Wasik *et al*. 2021). Our genotyping method takes advantage of the unique HS population structure caused by interbreeding 8 inbred founders. Because the founders are fully sequenced, we are able to construct a high-quality reference panel for HS rats, which enables us to achieve highly accurate imputation for their genotypes even with low read coverage (0.21× mean ddGBS and 0.27× mean lcWGS).

Others have reported a similar genotyping strategy of using GBS or lcWGS alone and imputation for AIL and CFW mice (Nicod *et al*. 2016; Parker *et al*. 2016; Gonzales *et al*. 2018). Nicod *et al*. (2016) used lcWGS sequence data with STITCH imputation on CFW mice. Parker *et al*. (2016) used GBS sequence data with IMPUTE2 on CFW mice. Gonzales *et al*. (2018) used GBS sequence data with BEAGLE on AIL mice. Their estimated genotype concordance rates were 98.1, 97.0, and 96.96% respectively compared to the MegaMUGA array. Our previous work of using ddGBS and imputation (2 rounds of imputation: BEAGLE and IMPUTE2) to genotype HS rats was able to produce over 3.7 million SNPs with a concordance rate of 99.0% compared to a custom Affymetrix Axiom MiRat 625k microarray (Gileta *et al*. 2020). These 4 studies also included a variant calling step to identify candidate variants using either ANGSD or GATK before imputation. Our genotyping method described here does not require such a variant calling step, which reduces computation. Our method combines ddGBS and lcWGS sequence data and uses STITCH in conjunction with a fully sequenced founder reference panel to achieve genotype imputation. As a result, we achieve a high genotype concordance rate (>99.76%) compared to high-coverage (33.26× coverage) WGS.

Our genotyping method provides a robust method for genotyping HS rats by effectively imputing SNP genotypes from 2 different sequencing protocols without significant batch effects. Even with low read coverage, our method produced highly accurate genotypes. Our method is cost-effective due to the previously developed affordable ddGBS technique and low-cost lcWGS, which use commercially available library preparation kits and liquid-handling robots, improving throughput. Additionally, our method combines ddGBS and lcWGS sequences for genotype imputation, enabling old ddGBS genotyped rats to be analyzed in tandem with more recently genotyped HS rats.

The differences we observed in aligned sequence statistics between ddGBS and lcWGS (Fig. 2) reflect the different nature of the DNA sequences captured by 2 sequencing methods. Double restriction enzyme digestion limits ddGBS to only capture the DNA fragments near the enzyme cut sites, while random priming helps lcWGS capture DNA fragments across the genome randomly. Despite the differences in captured DNA fragments, the genotype concordances of imputed SNPs for both ddGBS and lcWGS are remarkably high, at 99.76 and 99.78%, respectively. This concordance demonstrates the strength of our pipeline in producing high-accuracy genotypes in HS rats, which provides a strong foundation for genetic studies in this population.

The GBS sequencing method was originally developed by Elshire *et al*. (2011) and modified to accommodate other species such as soybean (Sonah *et al*. 2013), rice (Furuta *et al*. 2017), oat (Fu 2018), chicken (Pértille *et al*. 2016; Wang *et al*. 2017), fox (Johnson *et al*. 2015), cattle (Donato *et al*. 2013), and mouse (Parker *et al*. 2016; Gonzales *et al*. 2018). Our lab modified GBS for use in HS rats (Gileta *et al*. 2020). In this study, we further improved our genotyping methods by harmonizing the previously produced ddGBS sequences and newly sequenced lcWGS sequences with commercial WGS technique in support of large-scale genetic studies. The principles of our genotyping method can be easily adapted for use in other populations, especially for those in which the founders are fully sequenced.

In summary, we developed a genotyping method for HS rats that is both cost-effective and high-throughput, yielding highly accurate genotypes. Our method can be readily applied to other species with minimal adjustments, forming a basis for conducting extensive genetic research in nonhuman species.

## Supplementary Material

jkae291_Supplementary_Data

## Data Availability

HS rats are available at https://ratgenes.org/cores/core-b/. Wet lab procedures are documented in protocols.io https://www.protocols.io/workspaces/cgord (spleen cutting: http://dx.doi.org/10.17504/protocols.io.36wgq7nryvk5/v1, DNA extraction: http://dx.doi.org/10.17504/protocols.io.8epv59reng1b/v1, normalization and randomization: http://dx.doi.org/10.17504/protocols.io.261genw5dg47/v1, library preparation: http://dx.doi.org/10.17504/protocols.io.j8nlkkm85l5r/v1, pooling and sequencing: http://dx.doi.org/10.17504/protocols.io.yxmvmnw29g3p/v1). Raw sequencing reads for ddGBS and lcWGS are available in NCBI SRA: PRJNA1022514. Eight HS inbred founder WGS raw reads are available in NCBI SRA: PRJNA487943 and PRJNA1048943. Eighty-eight selected HS rat WGS raw reads are available in NCBI SRA: PRJNA1076141. High-coverage WGS GATK genotyping pipeline code is available in Zenodo (https://doi.org/10.5281/zenodo.6584834) and GitHub (https://github.com/Palmer-Lab-UCSD/High-Coverage-WGS-GATK-Genotyping-Pipeline). High-coverage WGS DeepVariant genotyping pipeline code is available in Zenodo (https://doi.org/10.5281/zenodo.10027133) and GitHub (https://github.com/Palmer-Lab-UCSD/High-Coverage-WGS-DeepVariant-Genotyping-Pipeline). Genotyping pipeline and analysis code is available in Zenodo (https://doi.org/10.5281/zenodo.10002191) and GitHub (https://github.com/Palmer-Lab-UCSD/HS-Rats-Genotyping-Pipeline). Genotype data after quality control are available in UC San Diego Library Digital Collections (https://doi.org/10.6075/J0445MPC). Supplemental material available at G3 online.
